# The Medical Examination of Civilian Airmen

**Published:** 1920-08-07

**Authors:** 


### THE MEDICAL EXAMINATION OF CIVILIAN AIRMEN.

The International Air Convention held in 1919 decided that all candidates presenting themselves for licences as pilots, navigators, or engineers in any form of civilian air transport must be examined on the basis of a standard laid down by the Convention. The requirements include an investigation of the family and personal history, surgical, medical, eye, ear, vestibular, and nose and throat examinations, but for the present these are only stated generally, the details of the medical examination being left to the individual countries.

The British method of procedure has lately been published, and the standards prescribed are, with certain additions, very much those which were in force for the pre-war army. Various special tests, *e.g.*, reaction time, muscle tone, which were tried during the war, have been discarded, not because they were found to be valueless, but because it was decided that until further investigation had been made the data supplied by them might be misleading. In addition, it was felt that, so far as British candidates were concerned, a history of aptitude at sports requiring eye was of equal value. The Americans, French and Italians have, however, expanded and laid much emphasis upon these tests.

Importance is attached to the necessity for dealing with the medical examination as a whole, and not rejecting a candidate because he fails in any one test, since the failure may be due to some purely temporary cause. The aim of the examination is not to estimate flying ability but to accept only those who will make safe pilots. Especially is this necessary at the present time, when the majority of candidates are already skilled pilots but may be suffering from war or flying stress. Maintenance of this standard is to be ensured by the employment of medical officers trained for this purpose and by the six-monthly re-examinations being made by the same medical officer who conducted the original examination.

In more detail, careful inquiries are made into previous diseases or injuries, with especial reference to those which might leave sequelæ affecting the cardio-vascular and nervous systems. Investigations are also made into habits and family history for evidence which might point to nervous instability, and the consensus of expert opinion appears to be strongly in favour of those who smoke and drink little and who sleep well. Demeanour during the examination is also to be noted, and it is suggested that the impression made on a trained observer as to general physique will be of more value than accumulated details.

The additions made to an ordinary medical examination are directed towards discovering how duties at high altitudes and over long flights are likely to be performed. Thus the "vital capacity" and coefficients of physical efficiency are determined, and the tone of the abdominal muscles is noted. As might be expected, tests for the cardio-vascular system are exhaustive, and a definite "Exercise Tolerance" is laid down. The pulse rate is taken, first sitting, then standing; this is followed by the regulated exercise, which consists of placing one foot on a chair and steadily raising the whole body to the height of the seat five times in fifteen seconds, one foot being retained on the chair throughout. While still standing the pulse is counted in five-second periods, and return to normal should take place in about fifteen to twenty-five seconds, the increase of pulse-rate being about twenty-four. The above might with advantage be adopted as a routine in all medical examinations for Government service in cases where the ability of the heart to perform work is called into question, since the various methods now adopted for this valuable test vary considerably in their results. The "breath-holding" test is defined, and the reasons for ceasing are to be given, since subjects who suffer from marked disability at altitudes almost invariably give an abnormal answer. Thus, "I became giddy or dizzy," "Things went blurred," "I began to feel squeamish," are regarded as not normal and are to be noted. The ophthalmic examination is of a high standard with a minimum distant visual acuity of 6/6 for each eye tested separately. But not enough stress is laid on tests for hemeralopia, *i.e.*, twilight vision, and for colour vision, although these must be of equal importance in aerial as in maritime navigation. A careful aural and nasopharyngeal examination is also made, and it is recommended that any minor defect should be remedied before the candidate is accepted.

It will thus be seen that so far as British pilots are concerned, a sound examination with a high standard has been prescribed. Whether this standard can be maintained is another question. There is no doubt that the physique of the nation has deteriorated, and that there is a general tendency towards being "high strung." Neither of these two factors make for the good average, and it may be difficult to obtain the numbers necessary to assure an adequate supply of pilots for the increasing needs of aerial transport. It is not obvious, should this occur, in what particulars the standard laid down could be safely lowered, but it would appear probable that for some years, at any rate, the examination cannot be too exacting.

